# Lived Experience of Parastomal Bulging: A Mixed Methods Study

**DOI:** 10.3389/jaws.2024.12478

**Published:** 2024-03-28

**Authors:** Julie Munro, Gill Hubbard, Will Goodman, Rebecca Beeken, Raymond Oliphant

**Affiliations:** ^1^ Centre for Rural Health Sciences, University of Highlands and Islands, Inverness, United Kingdom; ^2^ School of Health Sciences, University of Dundee, Dundee, United Kingdom; ^3^ School of Medicine, University of Leeds, Leeds, United Kingdom; ^4^ Colorectal Unit, Raigmore Hospital, NHS Highland, Inverness, United Kingdom

**Keywords:** stoma, hernia, self-management, parastomal hernia, parastomal bulging

## Abstract

**Aim:**

This United Kingdom study aimed to explore people’s experiences of living with, and self-managing parastomal bulging.

**Methods:**

Seventeen people were interviewed and 61 people completed an online survey.

**Results:**

Parastomal bulging has a detrimental impact on quality of life including a negative impact on stoma function, daily activities, body image, physical intimacy, and socialising; access to specialist information and support for addressing the problem of bulging was inequitable; support garments were the most common self-management intervention; there was confusion about what exercise would be beneficial or how being active would help in terms of parastomal bulging self-management; peer support is no substitute for high quality specialist support.

**Conclusion:**

People need equitable access to information and support to self-manage and treat parastomal bulging. Research about other types of self-management interventions, for example, exercise is required so that people do not have to rely solely on support garments to self-manage parastomal bulging.

## Introduction

Bowel stomas are created when parts of the bowel or rectum are removed due to surgery, or are no longer functioning due to disease. The remaining healthy bowel is then diverted through the abdominal wall where it discharges body waste into an externally-attached pouch. Such exposure of a previously hidden organ and elimination process profoundly changes a person’s physical and emotional wellbeing. In the United Kingdom, approximately 180,000 people live with a stoma, with worldwide data suggesting over a million ostomates in the United States, and China, and over 700,000 in Europe [1–3]. People with a stoma report numerous issues in relation to their quality of life (QoL) including leaks, appliance issues, and risk of developing parastomal bulging/hernia [4]. Parastomal bulging is a common problem following stoma formation [5, 6], with prevalence estimates over 30% by 12 months, 40% by 2 years and 50% or higher at longer duration of follow up [7]. People can have a bulge around the stoma but some bulges do not correspond to a hernia, which is an abnormal protrusion of the contents of the abdominal cavity through the abdominal wall defect created during placement of a colostomy, ileostomy or ileal conduit stoma [8]. From a patient perspective, lived experience of parastomal bulging and hernia may be indistinguishable. A large-scale survey in the Danish stoma population found that the reduction in QoL for those living with parastomal bulging was substantial and sustained [9]. Yet, despite parastomal bulging being a common and debilitating problem, research into people’s experiences of living with and managing parastomal bulging is rare.

Self-management of a parastomal bulge and support from healthcare professionals are priorities because it is unclear if progression of parastomal bulging can be stalled or prevented [10, 11]. Parastomal bulging can be repaired surgically but with reported recurrence rates of 30%–76% [12], patients are often left dealing with repeat parastomal bulging and further issues as a result of more surgery. Given the risks of further surgery and high risk of recurrence after repair, it is important that people with parastomal bulging are given effective support, help and guidance to self-manage parastomal bulging without surgical intervention. Current guidelines only offer surgery as an option for parastomal bulging treatment and offer advice on prevention [13]. Alternative self-management options are not part of current advice or guidelines in the United Kingdom. Given the paucity of research for non-surgical treatment options, this study aimed to explore people’s experiences of living with, and self-managing parastomal bulging. Self-Management involves helping a patient to understand their condition and to develop appropriate self-management behaviours [14]. Good self-management can lead to improved quality of life and a better adjustment to living with a stoma [15].

This study aimed to explore people’s experiences of living with, and self-managing parastomal bulging. The findings may be used to identify gaps in information and support and inform the development of a self-management intervention to improve QoL. In this paper we use the term parastomal bulging, which includes bulging and hernia.

## Materials and Methods

### Design

The study uses semi-structured one-to-one in-depth interviews of people living with parastomal bulging recruited from hospital out-patient clinic lists. Themes identified from analysis of interview data were investigated further by an online survey of people living with parastomal bulging recruited via social media.

### Ethical Approval

Full ethical approval was given for the project on October 21, 2021 by East Midlands—Derby Research Ethics Committee (Ref: 21/EM/0248) through the Integrated Research Application System (IRAS). Local site approvals for Participant Identification Centres were gained for both recruitment sites through HRA approval (England), and NHS Research and Development Management approval (Scotland).

### Patient Advisory Group

The Patient Advisory Group has engaged in our stoma and exercise feasibility research since 2017. In this study, the PAG advised on the project design, interview schedule and survey questions.

### Eligibility Criteria

People were eligible to participate if they were 16 years+ (in the United Kingdom, for the purposes of research, people 16 years old and over are adults [16]); ≥2 months post stoma formation surgery for bowel disease (e.g., Inflammatory Bowel Disease, colorectal cancer) with a colostomy or ileostomy who perceived that they have parastomal bulging or who have a clinical diagnosis of a parastomal hernia. People with previous hernia repair were also included.

### Recruitment

#### Interviews

Two United Kingdom hospitals were used to recruit participants from an outpatient clinic list. Potentially eligible patients were given written information about the project from a clinician. Verbal consent to be contacted was obtained by a clinician. Participant contact details were provided to the research team. Contact was then made by email/telephone by a researcher who confirmed eligibility, provided more information about the project and obtained further verbal or written consent from participants for their participation. An interview was arranged to take place online using Microsoft Teams, or Zoom depending on participant preference. Further verbal consent was obtained prior to each interview.

#### Survey

Social media was used to disseminate an advertisement about the project and invite those who were eligible to complete an online questionnaire. The research team shared the survey with relevant charity organisations to disseminate with their subscribers and followers. Participants completed an online consent form prior to completing the survey.

### Sampling

#### Interviews

How many participants are required for qualitative research remains equivocal [17]. Qualitative samples must be large enough so that all of the perceptions that might be important about experiences of living with and managing parastomal bulging are uncovered, but not too large so that data becomes repetitive and, eventually, superfluous. We aimed to use purposive sampling to recruit 20 participants with different types of stoma (ileostomy, colostomy), diagnoses (colorectal cancer, IBD) gender and age. The method of ‘data saturation’ [18] was used to determine the final sample size, which led to the final number of participants (n = 17) being slightly lower than our original target of 20 participants.

#### Survey

A convenience sample was used to recruit participants. The survey was designed to gather descriptive data about experiences of parastomal bulging. It was not statistically powered.

### Data Collection

#### Interviews

A semi-structured interview schedule with open-ended questions was developed by two members of the research team (JM, GH) and the Patient Advisory Group and included the following topics for discussion: 1) diagnosis of parastomal hernia, 2) experiences of parastomal bulging progression, e.g., Did bulging change over time?, 3) medical treatment and decision-making e.g., experience of help-seeking for parastomal bulging, 4) self-management e.g., use of support garments, 5) impact on QoL, e.g., impact on daily activities. JM conducted the online interviews; a female researcher with experience in qualitative methods and interviewing this patient population. There was no prior relationship between the interviewer and participants. Online qualitative interviewing methods are increasingly popular and work well using a structured interview approach [19]. Interviews lasted approximately 43 min. With participant’s permission, the interview was audio-recorded.

#### Survey

Two validated questionnaires relevant to investigating themes identified from analysis of interview data were used. The Patient Activation Measure is a validated questionnaire designed to measure the knowledge, skills and confidence that a person has to manage their own wellbeing (patient activation) [20]. Self-efficacy, which measures one’s expectations regarding the ability to perform some specific task was assessed using the 28-item stoma-related self-efficacy scale [21]. Other survey questions were about self-management for parastomal bulging with nine response options (e.g., support garments, staying active) and perceptions of these self-management approaches working with a yes/no response. Participants were also asked to rank in order importance five types of support for managing parastomal bulging (e.g., access to stoma nurse, support garments).

### Data Analysis

#### Interviews

The data from the audio-recorded interviews were anonymised by giving participants a study code, and by removing any reference of places, names, or NHS Trusts from the transcription before analysis. The Framework method, adopting thematic analysis, which is a rigorous method providing a structure within which qualitative data are organised and coded thematically, was used to analyse data by a member of the research team (JM, WG) [22]. Transcripts were read to gain an overall sense of the themes that were emerging from the data. Common words, phrases and sentences that conveyed a theme, or feeling, or experience were identified. The data were coded and categorised while new data was added through the iterative process and looking for a level of data saturation to ensure important issues were not missed. The comparison process was ongoing while new interviews were held and analysed to look for new emerging themes. The process provides a level of coding for the data produced which allows for themes to evolve around the data being collected rather than focusing on a fixed question.

#### Survey

All responses were summed and reported descriptively. For the Patient Activation Measure, raw data was entered into the scoring algorithm to produce a PAM score along an interval-level scale from 0–100 that corresponds to one of four levels. The four levels of the measure demonstrate levels of activation to engage in active self-management: Level 1—Disengaged and overwhelmed, 2—Becoming aware but still struggling, 3 –Taking action, Level 4—Maintaining behaviour and pushing forward. For the stoma self-efficacy scale, the score range was 28–140; the scale contains the Care Self-efficacy, and social self-efficacy all scored on a 5 point scale. <65 indicates low self-efficacy; 66–102 moderate self-efficacy; >103 high self-efficacy.

## Results

### Interviews

Seventeen people with parastomal bulging were interviewed and included six females and 11 males; the age range was 28–72 years. Seven participants had been treated for bowel cancer, five for inflammatory bowel disease, and five for other conditions (e.g., physical trauma, diverticulitis). The following results represent the most common themes that emerged. There are six themes and within the theme QoL there are four sub-themes. Quotations supporting themes identified are available in Table 1.

**TABLE 1:** Participant quotes from interview under thematic analysis headings.

Theme identified	
Physical changes	“The hernia … the hernia first of all, that happened slowly, and it just grew over a very long period of time, and it was … you know, it was just quite a while, “oh, yes, the surface is slightly raised, it’s becoming more and more domed,” and that probably happened, I do not know, perhaps gradually over 2 years maybe.”
	“In my mind, I’m thinking back to when COVID started, I had a ridiculous cough and sneeze that I could not get rid of, and I think I’ve coughed myself into a hernia. I think I coughed and sneezed so much”
	“Yeah. I struggle to put my socks on. I tend not to wear lace-up shoes anymore….that’s a bit … quite a big thing. Sort of physically, it’s … it’s mobility. It has a big impact on mobility, and also, you know, like trying to get out and play with my grandson and things like that, or if I want to lift him up to carry him, all that kind of thing is hard”
Quality of Life Social anxiety	*‘Oh, absolutely, yeah. Yeah, totally. Totally. Okay, 2 years, I do not go many places anymore, I really do not, because, you know, it’s very difficult to manage in … in that respect, it’s difficult to manage in how am I going to dress’*
	‘*It’s awful, I … I mean, I think, you know, I’ve probably become quite reclusive as a result of this. I tend not to accept too many things … I find it difficult … we had one wedding that we had to go down to, and it was just … it was just a dreadful experience. And I did have to change my bag when I got to the hotel. I … I wear a waistcoat when I go out to cover the bulge. But, yeah, it’s not … from that side of things, socialising, nah, I tend to avoid it.*’
	*And also I’m … I’m conscious that sometimes I can smell it a little bit; if I have not got the bag on quite right and there’s a bit of a … it’s not laying so flat, and if it’s playing up, I can smell it, oh, I do not know. It’s … it’s a mindset, is not it, really?*
Relationships	“Intimate-wise, my husband’s never looked at it, he does not want to know.”
	“I have ten grandchildren ….and it’s very difficult, you know, when someone goes to grab you or hug you…”
	“like trying to get out and play with my grandson and things like that, or if I want to lift him up to carry him, all that kind of thing I avoid’
*Daily activities*	“I think I’ve got this idea in my head that if I do anything at all, I’m going to muck it up and I’m going to end up in the position that I was the first time round. So, bending down, which I know is not a risk, I do not think it is a risk to … for parastomal hernia, but bending down, probably….But certainly lifting anything at all, even lifting a little bit. One thing I’ve noticed is I’ve got quite a … I think I’m conditioning my muscle mass has probably gone down quite a bit because I’m not doing as much as I was doing. And I’ve also noticed in the last month I’ve gained a lot of weight.”
Body Image	“It just makes me feel like a freak. You know, normal people do not walk around with their bum on their stomach. Sorry to put it like that, but that’s how I feel.”
	‘*Yeah. I … I know, I just did not understand that at the time, sort of Scottish man thing that you just put up with it. But, yeah, it is actually if I’m being absolutely honest, it does piss me off, and I cannot bear to look at myself in the mirror or anything like that, I just see the bullet hole on that side, a great big bulge on that side, hmm, yeah, it’s not … it’s not how I imagine myself to be*.’
Access to support	“Oh, and how I feel for people who do not have that support, and I’ve experienced both—my local stoma nurse was rubbish, quite honestly, and I asked my GP to transfer me back to [another hospital] because I had absolutely zero confidence in her, her knowledge…”
	“I have not seen the stoma nurses for a couple of years, I used to see them quite regularly because I had a lot of granulomas around the … the stoma, and I was going every one to 2 months to have the treatment on them. And really to no effect at all. And then the stoma nurse I was seeing retired suddenly, and then it just became more difficult to get an appointment, and then COVID hit”
	*“I do not know who should be giving me advice, I do not know who to turn to*”
Treatment options	*‘That’s … that’s the thing. I mean, my surgeon will not repair a hernia if it’s not bothersome. If it’s not causing pain, if it’s not obstructing you, he will not do it. But that’s only … that’s only a small part of it. It’s all about prevention. And also being able to manage it’*
Support Garments	*‘Yeah. I detest the … the vests and the belts. I really do not like them….I reluctantly wear my garments, but I wear it because … to cover my stoma bag popping out, peeping out at the end. I know that it will keep the stoma bag in, not because I feel it’s doing me good, I … I tend to get really bad leaks when I’m wearing my support bags, so it’s almost like it’s keeping everything in. It’s not allowing the stoma to function as it normally would, and then all of a sudden it just bursts through. I mean, I think the only times that I’ve had to change my bag, like in a car, which is the most horrible experience, is because I’ve had the support garments on’*
Intervention options	*‘Well, I do try to do some exercise, but I’m wondering whether I’m not helping myself. I do not wear a support belt, but I’m not sure whether I should be. All those things, really. I do not feel. I do not feel I know….’*
	*‘I’ve got a complete exercise booklet, it all comes from the* [*charity*] *people, but they gave me some in hospital, and when I came home and I was reading the Tidings magazine, I ordered some more as well. I’m not sure what I should be doing with any of it.’*

#### Physical Changes—Appearance and Function

For those reporting gradual onset, parastomal bulging seemed to have been apparent since stoma formation surgery, with slow worsening (e.g., pain, enlarging) over a period of months/years. For the few who experienced sudden onset of bulging, there was a particular moment or short time period in which bulging appeared. This was often due to coughing or sneezing. One participant reported that bulging had appeared on an old stoma site and then on newly sited stoma positions resulting in two parastomal bulges. The physical restrictions of having a large bulge were shared and included not being able to bend over properly to tie laced shoes, and on general mobility. When prompted about the effect of parastomal bulging on the function of their stoma, participants referred to bag adhesion issues and resulting leaks, being more prone to blockages due to change in shape, and also issues with emptying due to the shape of their bulge.

#### Quality of Life

##### Social Anxiety

Many participants discussed the role that their bulge had on their social lives, including anxiety about it being noticeable and a perception that they might smell. Some participants avoided socialising altogether due to low body image, or fear of leaks. Changes in the way people chose to dress to hide or disguise their bulge was common.

##### Relationships

Some participants reported difficulties with physical intimacy including difficulties discussing sex and intimacy with their partner. The bulge made the stoma and abdomen more ‘abnormal’ to look at and this had a detrimental effect on being physically intimate. The bulge affected family relationships as people were more aware of being hugged, and were fearful of, or completely avoided, playing with children/grandchildren in case of pain or worsening the size of the bulge. This avoidance style behaviour, associated with anxiety and fear, is echoed in other themes and sub-themes.

##### Daily Activities

Fear of bulging and bulging itself was a cause for anxiety and impacted daily activities. Perceptions of the bulge worsening and ‘doing the wrong thing’ was a recurring theme. There was fear of moving and not lifting ‘*anything heavier than a kettle*’ even several years after stoma formation surgery. Some patients were not advised on their risk of parastomal bulging following their stoma surgery. Bulging also detrimentally affected abilities to perform daily activities that involved bending such as putting on socks and shoes.

##### Body Image

Some participants discussed their relationships with their body and how they perceived it in its current form and body image was universally low. It was difficult to distinguish between the effect of the hernia, or the combination of the stoma itself and the hernia appearing later. Strong emotive words were used in some instances to convey the depth of feelings involved.

#### Access to Healthcare Support

Some participants felt well informed about their risk of parastomal bulging, whereas others remarked that they had never been advised that bulging might develop. Consistently, even for those who felt they were fairly well informed there was an expressed need for more information. Living with a stoma and parastomal bulging is a long-term condition requiring regular self-management and support from healthcare professionals. Throughout the interviews it was clear that there were variations in the level of care and support being provided from healthcare professionals. Some participants reporting excellent follow up care following surgery and direct access to their stoma care nurse, while others felt they had been left with little to no follow up care to manage their stoma and parastomal bulging. Commonly patients reported the delay in being measured for, and receiving abdominal support garments. The COVID-19 pandemic was often given as a reason for delays by healthcare professionals, and by patients. The complexity of stoma care and industry and supplies companies, and the employment of stoma nurses were openly discussed with a high level of awareness and understanding of this within this sample of patients. This came with some confusion about who to contact in the first instance for support: a surgeon, stoma care team in the hospital, community stoma nurse, GP, supplies companies, etc. One patient shared that he often heard the term ‘lost ostomate’ for people who had been lost to follow up in the complexities of the system. But consistently the importance of having clinical support from a stoma nurse, and knowing how and when to access that support was a priority.

#### Healthcare Treatments

When discussing treatments for parastomal bulging all participants were aware of surgery as a possible intervention. Discussions focused mainly on failed repairs, and surgeons not offering or refusing repair surgery due to the nature of the bulge and the high recurrence rate. Many reported that they did not want any more surgery, particularly in relation to the risk of recurrence and issues with more abdominal surgery and the recovery involved. No participants explicitly mentioned options they had been presented with that did not include surgery for their parastomal bulging self-management, with the exception of support garments. So, despite surgeons being open about risks and failure rates of repair surgery, participants were not provided with non-surgical alternatives at the time of these discussions within their healthcare team. Reasons by participants for not accepting surgical repair included bulging not affecting stoma function, previous failed repairs, anxiety over further surgery, bulge being too small, and original surgery already siting a mesh reinforcement at the stoma site.

#### Support Garments

Support garments were the main and often only self-management behaviour that participants used. Positive experiences included physical support, comfort and a feeling of control. Negative experiences with support garments included issues around delays in measurement and delivery after surgery; wrong sizing causing issues with bag functions or emptying; discomfort, leaks, pain, slipping down, poor bag emptying. Yet, even when participants attributed their support garment to negative experiences they still continued to wear the garment to support the bulge. A couple of participants indicated that they wear two support garments at one time for additional support. There was some confusion about when a garment should be worn, such as when physically active, lifting weights, carrying shopping, when lying down, or sleeping at night. A support garment was worn as a precaution. A recurring topic from almost all participants was the delay in support garment provision after surgery, the negative effect of the wait involved, and the lack of physical support for the stoma area post-surgery.

#### Interventions for Self-Management

Participants were asked what they felt was missing from their own supportive care, and what a potential intervention on parastomal bulging self-management might include for their personal needs. The ‘need for information’ was the overwhelming feedback, with no specific mode of delivery taking preference in terms of online, app, hard copies, videos, etc.,—all were deemed suitable. Information topics were wide ranging and included: support garment fitting; ranges of appliances available that might be better for herniated stomas, safe exercises pre-and post-surgery, weight management advice, and mental health support. There was brief mention of some supportive care pre-surgery, but there was no awareness that there may be many benefits to this type of approach. There was an understanding that being physically active was beneficial but there was confusion about what exercises would be beneficial or how being active would help in terms of parastomal bulging self-management.

Some participants welcomed peer support but there was no consistent feedback on how this might be delivered with mention of local support groups, online social media groups and charity input. Perceived benefits of peer support included mental health, ‘tips’ on managing daily challenges of ‘thickening output’, ‘bags that fit around the bigger lumps’, and managing and tidying up after recurrent leaks.

### Survey

Sixty-one people completed the online survey. Table 2 shows the demographic characteristics of those who completed the survey.

**TABLE 2:** Participant characteristics.

Variable		n
Sex	Male	23 (40%)
	Female	32 (55%)
	Prefer not to say	3 (5%)
Age		Mean 56 (min 33: max 88)
Type of stoma	Colostomy	27 (46%)
	Ileostomy	30 (51%)
	Urostomy	1 (1.5%)
	Unsure	1 (1.5%)
Permanent/temporary	Permanent	53 (90%)
	Temporary	6 (10%)
Reason for stoma	Bowel/Rectal cancer	26 (43%)
	IBD	19 (32%)
	Diverticulitis	2 (3%)
	Physical injury/trauma	1 (2%)
	Other	12 (20%)
Emergency/elective surgery	Emergency	18 (31%)
	Elective/waiting list	35 (59%)
	Unsure	6 (10%)
Length of time with bulging	0–6 months	6 (10%)
	7–12 months	5 (9%)
	13–18 months	8 (14%)
	19–24 months	5 (9%)
	25–36 months	9 (16%)
	37–48 months	5 (9%)
	More than 4 years	19 (33%)
Parastomal bulging	First occurrence	45 (79%)
	Recurrence after repair/re-site of stoma	12 (21%)

#### Patient Activation Measure

Sixty percent of survey participants scored in the activated phases for self-management of parastomal bulging on the patient activation measure (PAM) (Table 3).

**TABLE 3:** Patient activation measure (PAM) level.

	N (%)
Level 1 Disengaged and overwhelmed	11 (20%)
Level 2 Becoming aware but still struggling	11 (20%
Level 3 Taking action	18 (32%)
Level 4 Maintaining behaviour and pushing forward	16 (28%)

#### Self-Efficacy

The mean total self-efficacy score was 97 (min 42:max 137; standard deviation 25). Eighty-eight percent of participants scored medium (n = 25, 43%) to high (n = 26, 45%) self-efficacy for stoma care; twelve percent (n = 7) scored low self-efficacy.

**FIGURE 1:**
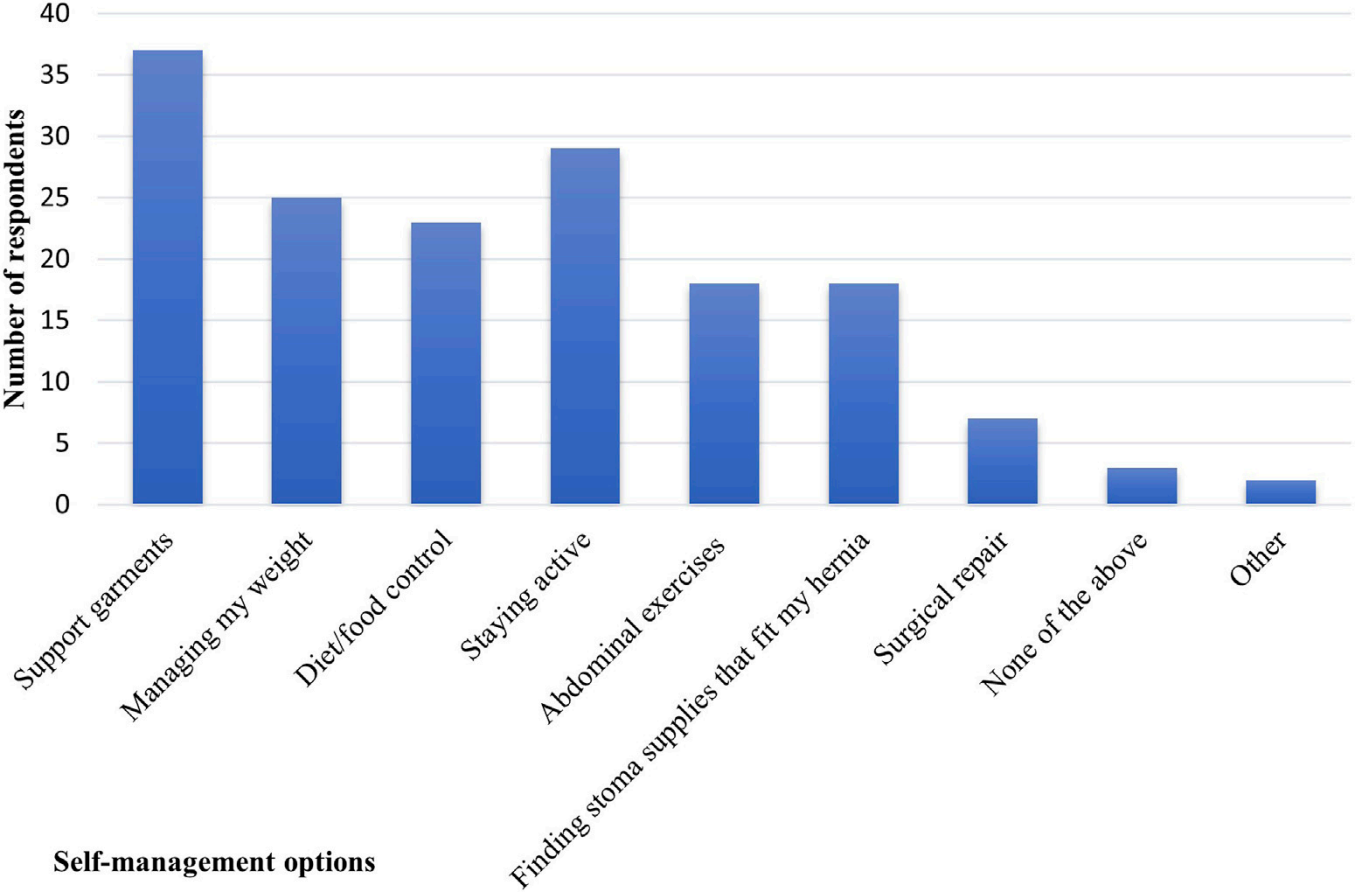
Frequency of use of parastomal bulging self-management choices. Multiple options were available for participants to choose (n = 61).

Support garments and staying active were the most commonly used self-management choices (Figure 1).

**FIGURE 2:**
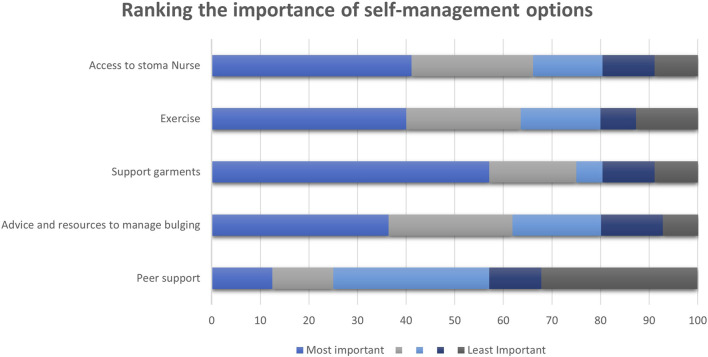
Stacked chart demonstrating the ranking from most important to least important in self-management options according to participants given in percentages.

Fifty-seven percent of participants ranked support garments as the most important type of support for managing parastomal bulging. This was followed by improved access to a stoma nurse and exercise advice (41% and 40% respectively) as shown in Figure 2.

## Discussion

There are few studies that have explored people’s lived experience of stoma and we believe that this is the first study to explore lived experience of parastomal bulging. The study adds new insights about the impact of parastomal bulging on QoL, perceptions of information and support, and self-management of parastomal bulging. The qualitative interviews show that parastomal bulging is detrimental to QoL. Parastomal bulging had a negative impact on stoma function, daily activities, body image, physical intimacy, and socialising. Behaviours such as avoiding social events, limiting contact with family, and fear of moving are all suggestive of avoidance coping strategies rather than of abilities to self-manage [23]. The study corroborates the findings of research about QoL in people with a stoma such as two cross-sectional studies which found that QoL was worse in those with a parastomal bulge compared to those without bulging [9, 24], and adds to a body of evidence about the negative impact of stoma on body image and sexual activity [25, 26].

The study suggests that people with parastomal bulging are likely to be receptive to self-management interventions. They did not believe that surgical repair was a panacea because they acknowledged for instance, that there was a risk of recurrence. Yet, participants in this study were not provided with non-surgical alternatives to addressing the problem of parastomal bulging. Lack of other types of intervention may be linked to another key finding from the study which is unevenness in access to information and support about the risk of parastomal bulging and how to manage bulging if it occurs. Some of the delays in access to support for parastomal bulging were attributed by participants to the COVID-19 pandemic which impacted all NHS services and NHS staff [27]. Nonetheless, delays occurred before, during, and after the pandemic and this study adds further weight to a recent United Kingdom Delphi study that reached consensus on the need for improved information for people with a stoma and concluded that access to specialist support should be equitable and consistent across the country [28]. Moreover, the study highlights that peer support, while welcome, is no substitute for high quality support and information from stoma care specialists.

The study found that the most important parastomal bulging self-management intervention used by participants is support garments. Support garments are not only invaluable for self-managing bulging but have been found to be used in the self-management of body complications, appearance, function and sensations [29]. Yet, support garments and underwear previously available to all stoma patients free of charge are now only prescribed to those with a clinical need (e.g., for existing hernia support [30]) due to lack of strong evidence for their efficacy in lower risk of herniation. Emphasis by participants on support garments however, may reflect lack of information about alternatives such as exercise to self-manage parastomal bulging. The study found that there was confusion about what exercise would be beneficial or how being active would help in terms of parastomal bulging self-management.

### Limitations

The main limitation is that the survey conducted as part of the study had a small convenience sample. The majority of participants in the survey were in the activated phases for self-management of parastomal bulging and had high levels of stoma care self-efficacy. This means that the study may not have captured the lived experience and perceptions of people with parastomal bulging who are not self-managing and have poor self-efficacy.

### Conclusion

People need equitable access to information and support to self-manage and treat parastomal bulging. Research about other types of self-management interventions, for example, exercise is required so that people do not have to rely solely on support garments to self-manage parastomal bulging.

## Data Availability

The data that support the findings of this study are available on request from the corresponding author [JM]. The data are not publicly available due to restrictions due to information that could compromise the privacy of research participants.

## References

[1] Hub EoENCP. StoMap Programme Baseline Report 2019. United Kingdom: NHS East of England (2019). Available from: https://www.eoecph.nhs.uk/Files/Integrated%20Care/StoMap%20Baseline%20Report%20FINAL.pdf (Accessed June 13, 2023).

[2] Carlsson E Berndtsson I Hallén A-M Lindholm E Persson E. Concerns and Quality of Life Before Surgery and During the Recovery Period in Patients With Rectal Cancer and an Ostomy. J Wound Ostomy Continence Nurs (2010) 37(6):654–61. 10.1097/WON.0b013e3181f90f0c 21052026

[3] Goodman W Downing A Allsop M Munro J Taylor C Hubbard G Quality of Life Profiles and Their Association With Clinical and Demographic Characteristics and Physical Activity in People With a Stoma: A Latent Profile Analysis. Qual Life Res (2022) 31(8):2435–44. 10.1007/s11136-022-03102-5 35217962 PMC9250477

[4] Hubbard G Taylor C Beeken B Campbell A Gracey J Grimmett C Research Priorities About Stoma-Related Quality of Life From the Perspective of People With a Stoma: A Pilot Survey. Health Expect (2017) 20(6):1421–7. 10.1111/hex.12585 28675608 PMC5689233

[5] Husain SG Cataldo TE. Late Stomal Complications. Clin Colon Rectal Surg (2008) 21(1):31–40. 10.1055/s-2008-1055319 20011394 PMC2780194

[6] Randall J Lord B Fulham J Soin B. Parastomal Hernias as the Predominant Stoma Complication After Laparoscopic Colorectal Surgery. Surg Laparosc Endosc Percutan Tech (2012) 22(5):420–3. 10.1097/SLE.0b013e31825d36d7 23047385

[7] Antoniou SA Agresta F Garcia Alamino JM Berger D Berrevoet F Brandsma HT European Hernia Society Guidelines on Prevention and Treatment of Parastomal Hernias. Hernia (2017) 22:183–98. 10.1007/s10029-017-1697-5 29134456

[8] Smietanski M Szczepkowski M Alexandre JA Berger D Bury K Conze J European Hernia Society Classification of Parastomal Hernias. Hernia (2014) 18(1):1–6. 10.1007/s10029-013-1162-z 24081460 PMC3902080

[9] Krogsgaard M Watt T Danielsen AK Klausen TW Vinther A Gogenur I Impact of a Parastomal Bulge on Quality of Life - A Cross-Sectional Study of Patients From the Danish Stoma Database. Ann Surg (2019) 274:e1085–e1092. 10.1097/SLA.0000000000003743 31850997

[10] Donahue TF Bochner BH Sfakianos JP Kent M Bernstein M Hilton WM Risk Factors for the Development of Parastomal Hernia After Radical Cystectomy. J Urol (2014) 191(6):1708–13. 10.1016/j.juro.2013.12.041 24384155 PMC4156556

[11] Hussein AA Ahmed YE May P Ali T Ahmad B Raheem S Natural History and Predictors of Parastomal Hernia After Robot-Assisted Radical Cystectomy and Ileal Conduit Urinary Diversion. J Urol (2018) 199(3):766–73. 10.1016/j.juro.2017.08.112 28890392

[12] Jones H Rees M Aboumarzouk O Brown J Cragg J Billings P Prosthetic Mesh Placement for the Prevention of Parastomal Hernia. Cochrane Db Syst Rev (2018)(7). 10.1002/14651858.CD008905.pub3 PMC6513624 30027652

[13] ACPGBI Parastomal Hernia Group. Prevention and Treatment of Parastomal Hernia: A Position Statement on Behalf of the Association of Coloproctology of Great Britain and Ireland. Colorectal Dis (2018) 20(S2):5–19. 10.1111/codi.14249 30176120

[14] NHS England. Supported Self-Management Education Guide. United Kingdom: NHS England (2023). Available from: https://www.england.nhs.uk/long-read/self-management-education/ (Accessed November 14, 2023).

[15] Villa G Manara DF Brancato T Rocco G Stievano A Vellone E Life With a Urostomy: A Phenomenological Study. Appl Nurs Res (2018) 39:46–52. 10.1016/j.apnr.2017.10.005 29422176

[16] Health Research Authority. Research Involving Children: Health Research Authority (2018). Available from: https://www.hra.nhs.uk/planning-and-improving-research/policies-standards-legislation/research-involving-children/ (Accessed March 14, 2019).

[17] Cleary M Horsfall J Hayter M. Data Collection and Sampling in Qualitative Research: Does Size Matter? J Adv Nurs (2014) 70(3):473–5. 10.1111/jan.12163 24450874

[18] Francis JJ Johnston M Robertson C Glidewell L Entwistle V Eccles MP What Is an Adequate Sample Size? Operationalising Data Saturation for Theory-Based Interview Studies. Psychol Health (2010) 25(10):1229–45. 10.1080/08870440903194015 20204937

[19] Lobe B Morgan DL Hoffman K. A Systematic Comparison of In-Person and Video-Based Online Interviewing. Int J Qual Methods (2022) 21:160940692211270. 10.1177/16094069221127068

[20] Hibbard JH Stockard J Mahoney ER Tusler M. Development of the Patient Activation Measure (PAM): Conceptualizing and Measuring Activation in Patients and Consumers. Health Serv Res (2004) 39(4):1005–26. 10.1111/j.1475-6773.2004.00269.x 15230939 PMC1361049

[21] Bekkers MJ van Knippenberg FC van den Borne HW van Berge-Henegouwen GP. Prospective Evaluation of Psychosocial Adaptation to Stoma Surgery: The Role of Self-Efficacy. Psychosom Med (1996) 58(2):183–91. 10.1097/00006842-199603000-00013 8849636

[22] Smith J Firth J. Qualitative Data Analysis: The Framework Approach. Nurse Res (2011) 18(2):52–62. 10.7748/nr2011.01.18.2.52.c8284 21319484

[23] Audulv Å Packer T Hutchinson S Roger KS Kephart G. Coping, Adapting or Self-Managing–What Is the Difference? A Concept Review Based on the Neurological Literature. J Adv Nurs (2016) 72(11):2629–43. 10.1111/jan.13037 27272388

[24] Kald A Juul KN Hjortsvang H Sjodahl RI. Quality of Life Is Impaired in Patients With Peristomal Bulging of a Sigmoid Colostomy. Scand J Gastroenterol (2008) 43(5):627–33. 10.1080/00365520701858470 18415759

[25] Druss RG O'Connor JF Stern LO. Changes in Body Image Following Ileostomy. Psychoanal Q (1972) 41(2):195–206. 10.1080/21674086.1972.11926594 5021046

[26] Manderson L. Boundary Breaches: The Body, Sex and Sexuality After Stoma Surgery. Soc Sci Med (2005) 61(2):405–15. 10.1016/j.socscimed.2004.11.051 15893055

[27] Marinova P Marinova R. Innovation and Digital Nursing: Providing Continuity in Stoma Care to Patients During the Pandemic. Br J Nurs (2023) 32(16):S46–S48. 10.12968/bjon.2023.32.16.S46 37682766

[28] Aibibula M Burry G Gagen H Osborne W Lewis H Bramwell C Gaining Consensus: The Challenges of Living With a Stoma and the Impact of Stoma Leakage. Br J Nurs (2022) 31(6):S30–S39. 10.12968/bjon.2022.31.6.S30 35333550

[29] Hubbard G Taylor C Munro J Dames N Goodman W Oliphant R Experiences of Support Garments Following Bowel Stoma Formation: Analysis of Free-Text Responses in a Cross-Sectional Survey. BMJ Open Gastroenterol (2019) 6(1):e000291. 10.1136/bmjgast-2019-000291 PMC6577355 31275585

[30] Clyde NGGa. Support Garments for the Prevention and Management of Parastomal Hernias (2021). Available from: https://ggcmedicines.org.uk/blog/medicines-update/support-garments-for-the-prevention-and-management-of-parastomal-hernias/:NHSGreaterGlasgowandClyde (Accessed May 05, 2023).

